# Supplementary material for: Empirical methods for controlling false positives and estimating confidence in ChIP-Seq peaks
Source: BMC Bioinformatics. 2008 Dec 5;9:523. doi: 10.1186/1471-2105-9-523 (PMC2628906; doi:10.1186/1471-2105-9-523)
Supplement: Additional File 1 — A variety of html documents from the USeq web site detailing the available applications, their best usage, output file type descriptions, command line menus, etc. [file 1471-2105-9-523-S1.zip › USeqUserGuides/JavaDoc.zip!/JavaDoc/util/bio/parsers/package-frame.html]

util.bio.parsers


util.bio.parsers

Classes 
  
ADFDataBaseSplitter
  
BaseCount
  
BlastCompompositeGenerator
  
BlastParser
  
Cap3Parser
  
DrosGeneCollFileParser
  
Fasta
  
FetchGenomicSequences
  
FetchSeqsQualsByName
  
MultiFastaParser
  
ParseDecodeGenotypeData
  
QualityFileParser
  
RefSeqAnnotationParser
  
RipInparanoidResults
  
RipMGIOrthologyResults
  
ShiftPositionsInUCSCGeneLines
  
SynonymMatching
  
UCSCGeneLine
  
UCSCGeneLineComparator
  
UCSCGeneLineComparatorScore
  
UCSCGeneModelTableReader
